# Health-Related Quality of Life After Laparoscopic Nissen Fundoplication: A Long-Term Single-Center Cohort Analysis

**DOI:** 10.7759/cureus.92686

**Published:** 2025-09-19

**Authors:** Mauro Sousa, Maria João Samúdio, Luís Castro, Luís Miranda, Fernanda Quirino

**Affiliations:** 1 General Surgery, Unidade Local de Saúde de Santa Maria, Lisbon, PRT

**Keywords:** anti-reflux surgery, gastroesophageal reflux disease (gerd), health-related quality of life, laparoscopic nissen fundoplication, patient-reported outcomes

## Abstract

Introduction and objectives: Gastro-oesophageal reflux disease (GERD) significantly impairs quality of life, particularly in patients with persistent symptoms despite optimal medical therapy. Laparoscopic Nissen fundoplication (LNF) is considered the gold-standard surgical treatment for well-characterised, refractory GERD. This study aimed to retrospectively evaluate long-term health-related quality of life (HRQoL), patient satisfaction, and proton pump inhibitor (PPI) use following LNF in a single-centre cohort.

Methods: All consecutive patients undergoing LNF at our institution between January 2009 and December 2019 were retrospectively reviewed. Demographic data, medication use, reoperations, and satisfaction were collected. Symptom burden was assessed using an adapted version of the validated GERD-HRQL questionnaire, administered by a structured telephone interview. Pre- and postoperative scores were compared using the Wilcoxon signed-rank test, with significance set at p < 0.05.

Results: Sixty-eight patients were included (79.4% female, median age 66 years). Median follow-up was 5.5 years. Postoperative discontinuation of PPIs occurred in 53.7% of patients; those who continued therapy were mostly receiving concomitant ulcerogenic medication. Satisfaction with surgical outcome was reported by 84.6% of respondents. GERD-HRQL scores, assessed with the adapted instrument, improved significantly across most domains, including heartburn, regurgitation, and symptom-related sleep and dietary interference (all p < 0.001). Dysphagia showed a non-significant trend towards improvement (p = 0.084). Reoperation was required in two patients (2.9%), and no procedure-related mortality occurred.

Conclusion: LNF offers durable symptom relief, improved HRQoL, and high long-term satisfaction, with low reoperation rates. Persistent PPI use and the non-significant trend in dysphagia highlight the importance of realistic preoperative counselling.

## Introduction

Gastro-oesophageal reflux disease (GERD) is one of the most common upper gastrointestinal disorders, with a substantial impact on healthcare systems and patient quality of life [1]. Although the majority of patients respond to lifestyle modification and proton pump inhibitors (PPIs), a proportion continue to experience troublesome symptoms, develop intolerance to long-term therapy, or prefer a definitive treatment option [2].

Surgical intervention is well established in this context. Laparoscopic Nissen fundoplication (LNF), first introduced in 1956 and subsequently adapted to minimally invasive techniques, has become the most widely performed anti-reflux operation. According to international consensus and guideline statements, LNF is considered the gold-standard surgical treatment for GERD [3].

Beyond durability of symptom control, reoperation rates, and medication discontinuation, health-related quality of life (HRQoL) is a particularly relevant outcome in GERD. The disease affects not only physical symptoms such as heartburn and regurgitation but also daily functioning, sleep, diet, and social well-being. Patient-reported outcomes are therefore essential to evaluate the long-term benefits of surgery beyond objective physiological measurements [4].

The objective of this study was to retrospectively assess the long-term impact of LNF on HRQoL, patient satisfaction, and PPI use, using an adapted GERD-HRQL questionnaire [5,6] in a single-centre cohort.

## Materials and methods

Study design and setting

This was a retrospective cohort study including all consecutive patients who underwent LNF, with or without concomitant hiatal hernia repair, at the Hospital de Santa Maria, Lisbon, Portugal, between January 2009 and December 2019. All procedures were performed by a dedicated surgical team.

Eligibility criteria

**Figure 1:**
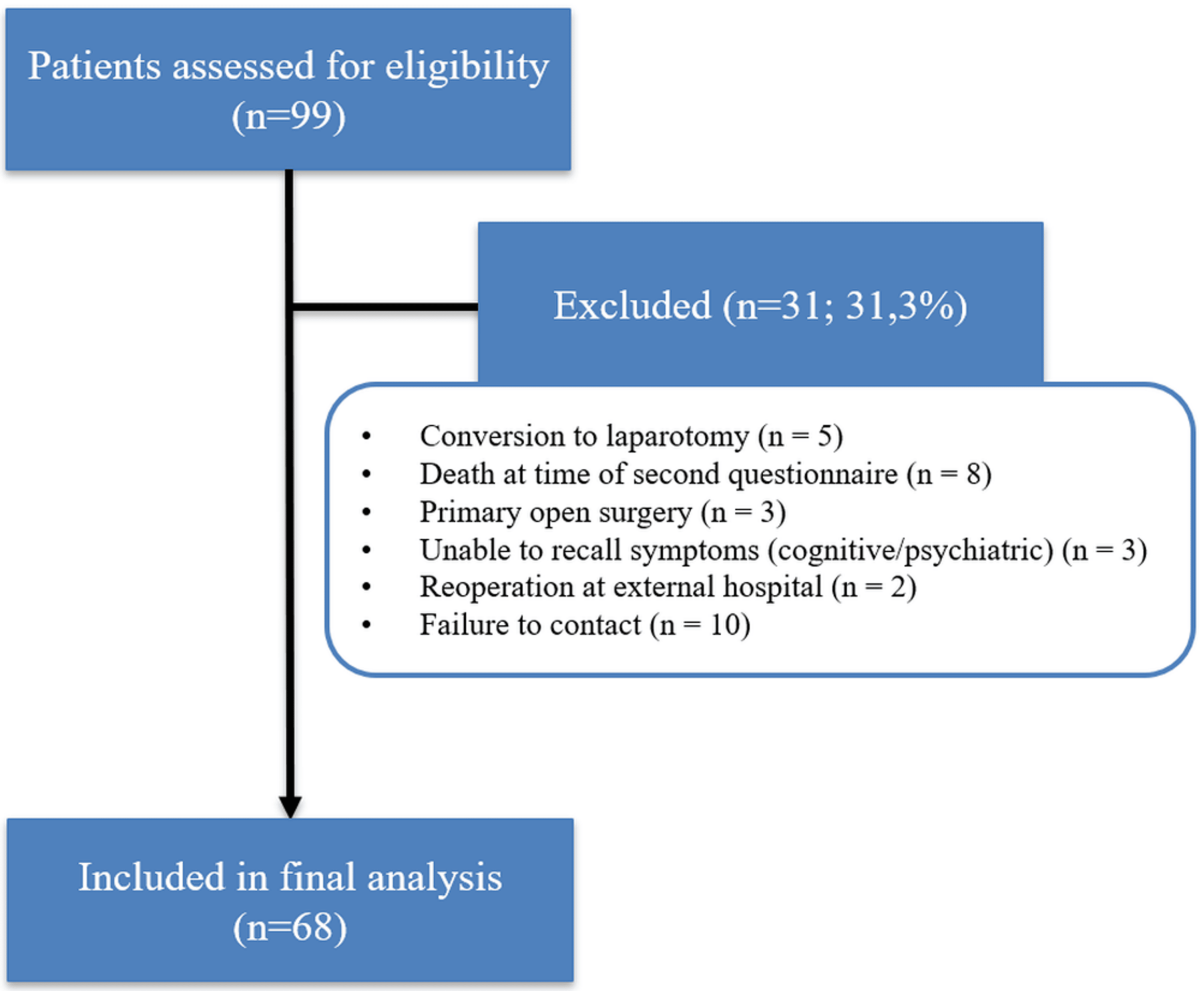
A flow diagram of patient inclusion and exclusion A total of 99 patients were assessed for eligibility; 31 were excluded for predefined reasons, leaving 68 patients for the final analysis.

Patients were eligible if they had GERD supported by typical symptoms plus objective confirmation on preoperative work-up (endoscopy and/or pathological acid exposure on 24-hour pH monitoring). In line with current practice, pH monitoring was omitted in cases of severe erosive oesophagitis on endoscopy (Los Angeles grade C/D). Exclusion criteria were predefined and are summarised in the flow diagram (Figure 1).

Participant flow

Of 99 patients initially identified, 68 were included in the final analysis. Thirty-one patients were excluded for the following reasons: conversion to laparotomy (n = 5), death from unrelated causes at the time of the second follow-up questionnaire (n = 8), primary open surgery (n = 3), inability to recall preoperative symptoms due to cognitive or psychiatric conditions (n = 3), reoperation at an external hospital (n = 2), and failure to contact despite multiple attempts (n = 10) (Figure 1).

Surgical technique

All procedures were performed laparoscopically following a standardised approach. The short gastric vessels were routinely divided to ensure a tension-free wrap. The distal oesophagus was mobilised for at least 3 cm into the abdominal cavity, and the hernia sac was dissected when present. A 360° floppy fundoplication was constructed without the use of a calibration bougie. Hiatal closure was systematically performed with three interrupted silk sutures. The majority of patients either had no hiatal hernia or a small type I (sliding) hernia, and in these cases, the hiatal defect was consistently closed with sutures only, without mesh reinforcement.

Outcomes and data collection

The primary outcome was change in disease-specific HRQoL, measured with an adapted GERD Health-Related Quality of Life (GERD-HRQL) questionnaire (Appendix A). This version comprises the original validated 10-item instrument [5,6] (items scored 0 to 5; lower scores indicate better symptom control) [6]. Secondary outcomes included patient satisfaction (0-10 global scale) and PPI use. Follow-up was conducted through structured, interviewer-administered telephone questionnaires. Preoperative GERD-HRQL scores were collected retrospectively by patient recall during these interviews, which is acknowledged as a potential source of recall bias.

Statistical analysis

Data were analysed using IBM SPSS Statistics (version 30.0, IBM Corp., Armonk, NY, USA). Continuous variables were tested for normality using the Shapiro-Wilk test. As most variables were non-normally distributed, results are presented as median (interquartile range, IQR). Comparisons between pre- and postoperative GERD-HRQL scores were performed with the Wilcoxon signed-rank test, with test statistics (Z) and two-tailed p-values reported. Statistical significance was set at p < 0.05.

Analysis of population and missing data

Paired pre- and postoperative comparisons were restricted to patients with both GERD-HRQL assessments (n = 68). A complete-case approach was used, and denominators are specified where responses were missing (e.g., patient satisfaction n = 65/68). No adjustment for multiple comparisons was performed, and p-values are interpreted as exploratory.

## Results

A total of 68 patients were included in the final analysis (Table 1). All patients completed both pre- and postoperative GERD-HRQL questionnaires, and patient satisfaction responses were available for 65 of 68 patients (95.6%). Of these, 79.4% (n = 54) were female. The median age was 66 years (range: 27-86 years), and the median postoperative follow-up duration was 5.5 years (IQR: 3.0-8.0 years). The reoperation rate was 2.9% (n = 2), and there were no procedure-related deaths.

**Table 1:** Characteristics of the study population Data are presented as n (%) or median (interquartile range, IQR). PPI: proton pump inhibitor

Variables	Results
Age, years (IQR)	66 (57-75)
Gender, female, n (%)	54 (79.4)
Postoperative follow-up duration, years (IQR)	5.5 (3.0-8.0)
Reoperation rate, n (%)	2 (2.9)
Perioperative mortality, n (%)	0
PPI use prior to surgery, n (%)	61 (91.0)
PPI discontinuation after the procedure, n (%)	36 (53.7)

Prior to surgery, 91% (n = 61) of patients were on PPIs. Following LNF, 53.7% (n = 36) were able to discontinue PPI therapy. Among those who continued PPIs (46.3%, n = 31), most were taking concurrent medications such as non-steroidal anti-inflammatory drugs (NSAIDs), corticosteroids, or other agents requiring gastric protection due to increased risk of peptic ulcer disease.

Patient satisfaction (Table 2) was high: 84.6% of respondents reported being satisfied with their surgical outcome (95% CI: 73.9-91.4%), 6.2% were neutral (95% CI: 2.4-14.8%), and 9.2% expressed dissatisfaction (95% CI: 4.3-18.7%). Patient satisfaction responses were available for 65 of 68 patients (95.6%). The median satisfaction score was 8 (on a scale from 0 to 10).

**Table 2:** Patients’ satisfaction level after surgery. Values are presented as percentages with 95% confidence intervals. Denominators are specified (n = 65/68 respondents).

Satisfaction Level	Frequency	Confidence intervals
Satisfied, n (%)	55 (84.6%)	95% CI: 73.9–91.4%
Neutral, n (%)	4 (6.2%)	95% CI: 2.4–14.8%
Dissatisfied, n (%)	6 (9.2%)	95% CI: 4.3–18.7%

Postoperative symptom improvement (Table 3) was significant across nearly all assessed domains. The GERD-HRQL domains are scored from 0 to 5, with lower values indicating better symptom control. Median scores decreased substantially in relation to heartburn (overall, positional, and postprandial), regurgitation, and symptoms interfering with sleep or diet (p < 0.001 for all). For dysphagia, scores showed a non-significant trend towards improvement (p = 0.084).

**Table 3:** Symptoms evaluation pre- and post surgery Domain-level GERD-HRQL results pre- and post-LNF; items 1–9 correspond to the adapted GERD-HRQL instrument [5,6], with the exception of the “gassy/bloating feelings” item, which was omitted. Items 10–15 represent an additional regurgitation block used in this adapted version. Values are median (IQR). Wilcoxon signed-rank test reported as Z and p values.

Symptom	Pre-surgery	Post surgery	Test value	p-value
Heartburn	3.5 (2.0–5.0)	0 (0–1.0)	Z=–6.545	<0.001
Heartburn when lying down	4.0 (2.0–5.0)	0 (0–1.0)	Z=–6.472	<0.001
Heartburn when standing	3.0 (1.0–4.0)	0 (0–0)	Z=–6.117	<0.001
Heartburn after meals	4.0 (2.0–5.0)	0 (0–1.0)	Z=–6.325	<0.001
Heartburn alters diet	3.0 (1.0–4.0)	0 (0–1.0)	Z=–5.339	<0.001
Heartburn disrupts sleep	2.0 (0.0–4.0)	0 (0–0)	Z=–5.190	<0.001
Difficulty swallowing	0 (0.0–1.0)	0 (0–1.0)	Z=–1.730	0.084
Pain with swallowing	0 (0.0–1.0)	0 (0–0)	Z=–2.831	0.005
Medication affects daily life	0 (0.0–1.0)	0 (0–0.0)	Z=–2.911	0.004
Regurgitation	2.5 (1.0–4.0)	0 (0–1.0)	Z=–5.892	<0.001
Regurgitation when lying down	2.0 (1.0–4.0)	0 (0–1.0)	Z=–5.682	<0.001
Regurgitation when standing	1.0 (0.0–2.0)	0 (0–0)	Z=–4.853	<0.001
Regurgitation after meals	2.0 (1.0–4.0)	0 (0–1.0)	Z=–5.761	<0.001
Regurgitation alters diet	3.0 (0.0–4.0)	0 (0–1.0)	Z=–5.085	<0.001
Regurgitation disrupts sleep	0 (0.0–1.0)	0 (0–0)	Z=–3.331	<0.001

## Discussion

This study shows that LNF provides significant and durable improvement in disease-specific HRQoL, with high long-term patient satisfaction and low reoperation rates. Nevertheless, nearly half of the patients continued to use PPIs, and dysphagia showed only a non-significant trend towards improvement.

Our results are consistent with previous long-term series of LNF, which also report durable symptom control, high satisfaction rates, and low reoperation rates [7-9]. Compared with these studies, our cohort is of moderate size but has a median follow-up of 5.5 years, which strengthens the reliability of the findings. Similar to prior reports, persistent PPI use remains common, reflecting not only ongoing reflux-related symptoms but also prescriptions for other indications such as gastric protection in patients requiring antiplatelet or anti-inflammatory therapy [10].

The non-significant trend towards improvement in dysphagia highlights the complexity of this symptom, which may persist even after technically successful surgery. Transparent reporting of this result avoids overstatement and aligns with recommendations on the importance of patient-reported outcomes in antireflux surgery [11].

This study has certain limitations. First, its retrospective design introduces the risk of recall bias, since preoperative HRQoL scores were collected retrospectively during follow-up interviews. As such, our findings demonstrate associations rather than causal relationships, and potential confounders (such as lifestyle modifications or the natural course of disease) cannot be excluded. Second, approximately 10% of the initial cohort could not be contacted, representing a potential non-responder bias. Third, as this was a single-centre study conducted at a tertiary referral hospital, the findings may not be generalisable to other settings with different patient populations or surgical expertise. Fourth, while the median follow-up was 5.5 years, the variability (range three to eight years) may mask late symptom recurrence. Finally, the absence of a control group prevents direct comparison with alternative surgical procedures or medical therapy. Persistent use of PPIs in nearly half of the cohort also represents a clinically relevant limitation that should be addressed in preoperative counselling. 

Furthermore, we used an adapted version of the GERD-HRQL, in which the original “gassy/bloating feelings” item was omitted and replaced by a six-item regurgitation block. While this adaptation reflects clinical practice and provides useful information, it has not been formally validated, which may limit direct comparability with studies using the original 10-item instrument.

Despite these limitations, our findings provide valuable long-term patient-reported outcome data, reinforcing the role of LNF as a safe and effective surgical option for appropriately selected patients with GERD.

## Conclusions

LNF is a safe, effective, and durable surgical treatment for patients with well-characterised, refractory GERD. In this single-centre cohort, the procedure was associated with sustained improvement in symptom control, enhanced HRQoL, and high long-term patient satisfaction. More than half of the patients discontinued proton pump inhibitors after surgery, and the need for reoperation was very low.

These findings highlight the value of LNF not only in reducing symptom burden but also in restoring overall patient well-being in selected individuals. Persistent PPI use and the non-significant trend regarding dysphagia underscore the importance of preoperative counselling, realistic expectations, and tailored postoperative follow-up. Future prospective, multicentre studies are needed to validate these results and further refine patient selection criteria and perioperative management strategies.

## Figures and Tables

**Figure 2:**
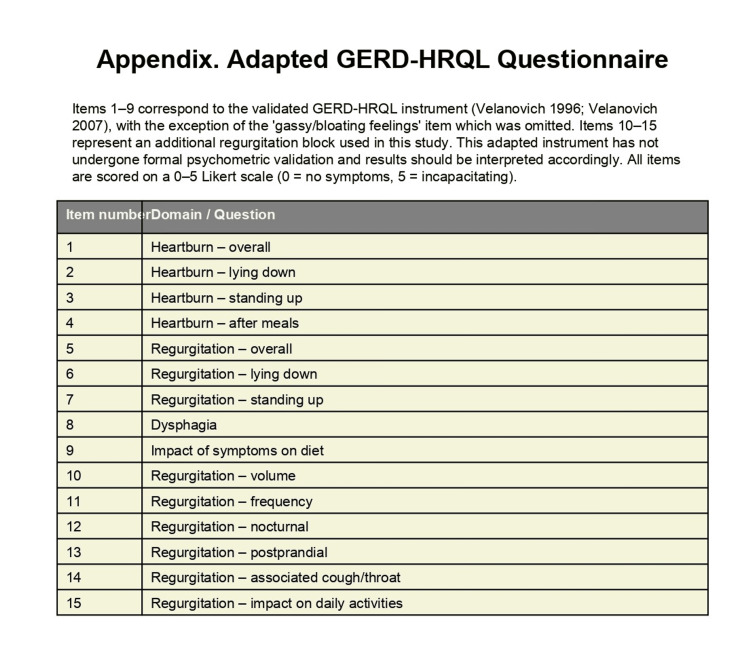
The adapted version of the validated GERD-HRQL questionnaire Source: [5,6]

## Appendices

Appendix A
